# Effect of Low-Load Long-Duration Stretch on Range of Motion in Post-Operative Knee Conditions

**DOI:** 10.7759/cureus.115430

**Published:** 2026-08-30

**Authors:** Anushka V Pawar, Trupti Yadav

**Affiliations:** 1 Department of Oncology Physiotherapy, Krishna College of Physiotherapy, Krishna Vishwa Vidyapeeth (Deemed to be University), Karad, IND

**Keywords:** joint contracture, knee range of motion (rom), knee rehabilitation, low-load long-duration stretch, postoperative knee stiffness

## Abstract

Background

Post-operative knee stiffness is a common complication following surgical fixation of fractures around the knee joint, leading to reduced range of motion, pain, and functional limitations. Early physiotherapy intervention is essential to restore mobility and improve function. Low-load long-duration stretch (LLLDS) is a prolonged stretching technique that enhances soft tissue extensibility and joint mobility with minimal discomfort. This study aimed to evaluate the effect of LLLDS on knee range of motion, pain, and functional outcomes in post-operative knee patients.

Objectives

To evaluate the effectiveness of low-load long-duration stretch on knee range of motion, pain reduction, and functional outcomes in post-operative knee conditions and to compare its effectiveness with conventional physiotherapy.

Methods

A randomized controlled experimental study was conducted at Krishna College of Physiotherapy, Karad, India. Thirty-six individuals were assessed for eligibility, of whom six were excluded before randomization. The remaining 30 participants were randomly allocated to the experimental group (n=15) and control group (n=15). Six participants were subsequently lost to follow-up or excluded after allocation, resulting in 24 participants (12 per group) completing the study. Participants aged 20-60 years who had undergone surgical fixation around the knee joint and completed six weeks of immobilization were included. They were randomly allocated to an experimental group (LLLDS with conventional physiotherapy) and a control group (conventional physiotherapy alone), with 12 participants in each group. Outcome measures included the Numerical Pain Rating Scale (NPRS), knee range of motion using a universal goniometer, and the Lysholm Knee Scoring Scale. Interventions were administered for four weeks. Paired t-test and unpaired t-test were used for statistical analysis.

Results

Both groups demonstrated statistically significant improvements in pain, knee range of motion, and functional outcomes following the four-week intervention (p<0.0001). The experimental group demonstrated lower post-intervention pain scores on the Numerical Pain Rating Scale (2.10±0.82 vs. 4.20±0.90; p=0.015), greater knee range of motion (106.33±2.93° vs. 103.50±2.68°; p=0.021), and higher Lysholm Knee Scoring Scale scores (88.75±6.40 vs. 84.10±6.85; p=0.039) compared with the control group. These findings suggest that adding LLLDS to conventional physiotherapy may provide additional benefits in reducing pain, improving knee mobility, and enhancing functional outcomes in post-operative knee conditions.

Conclusion

Low-load long-duration stretch combined with conventional physiotherapy is more effective than conventional physiotherapy alone in improving knee range of motion, reducing pain, and enhancing functional outcomes in post-operative knee conditions.

## Introduction

Post-operative knee stiffness (POKS) is a common complication observed after surgical fixation of bony injuries around the knee joint. Patients often experience different levels of stiffness following surgery, which can significantly affect joint mobility and overall functional performance. Compared with other joints, such as the ankle, elbow, and wrist, knee stiffness has a more pronounced impact on an individual’s daily functioning. While mild stiffness in the ankle or wrist may still allow satisfactory performance of routine tasks, stiffness in the knee can considerably limit occupational duties, activities of daily living, and recreational participation. The extent to which these functions are affected largely depends on the severity of post-operative knee stiffness present in the patient [1].

Abnormalities in the connective tissue structures surrounding the knee joint have been identified as major contributors to restricted passive range of motion (PROM). Evidence from gross anatomical, histological, and biomechanical studies suggests that both intra-articular and extra-articular tissues play a role in limiting joint mobility. Factors such as the formation of adhesions within the joint, stiffness of the periarticular joint capsule, and shortening of the surrounding skeletal muscles can all lead to reduced PROM. Together, these structural changes interfere with normal joint movement and contribute to the development of post-operative stiffness [2].

Several non-invasive therapeutic devices used in the management of post-operative knee arthrofibrosis are designed based on the biomechanical principles of creep and stress relaxation to improve joint mobility. Creep loading involves the application of a low-intensity, sustained force over an extended period, commonly delivered through springs or elastic components, to gently elongate shortened soft tissues. This gradual stretching helps increase flexibility and range of motion [3].

On the other hand, the stress relaxation principle focuses on positioning the joint at or near its end range and maintaining it in that position for a prolonged duration. Over time, the soft tissue structures adapt to the sustained stretch, leading to a gradual reduction in internal resistance and allowing improved joint movement. Both approaches aim to restore mobility by promoting tissue extensibility and reducing stiffness in the affected knee joint [3].

Research conducted on animals indicates that when muscles are immobilized in a shortened position, there may be a reduction in the number of sarcomeres arranged in series, which contributes to decreased muscle flexibility. In contrast, regular and prolonged stretching can lead to structural and morphological adaptations within the muscle, resulting in a sustained increase in tissue extensibility and improved range of motion [4].

Low-load long-duration (LLLD) stretching refers to the application of a gentle, sustained force over an extended period to promote elongation of connective tissue and improve joint mobility. When a low-intensity stretch is maintained for a prolonged duration, it facilitates the gradual lengthening of soft tissues and enhances tissue extensibility. This method of prolonged stretching is considered safer and more effective than high-intensity, short-duration stretching, particularly in the management of chronic contractures and post-operative joint stiffness. Sustained stretch can be achieved through the use of mechanical devices, weights, splints, or specific positioning techniques that help maintain the joint in a lengthened position. The effectiveness of this approach is based on the biomechanical principles of creep and stress relaxation of connective tissue, which allow tissues to gradually adapt and reduce resistance to stretch over time [5].

Building on this understanding of post-operative knee stiffness and the benefits of prolonged stretching, the present study aims to evaluate the effectiveness of LLLD stretching on knee range of motion in individuals following surgical fixation. Since post-operative stiffness can limit joint mobility and functional activities, this study focuses on determining whether sustained, low-intensity stretching can reduce stiffness and improve knee function. The findings are expected to support the use of LLLD stretching as an effective rehabilitation approach for enhancing recovery and functional outcomes after knee surgery.

## Materials and methods

Selection of subjects

The present randomized controlled experimental study was conducted at Krishna College of Physiotherapy, Krishna Vishwa Vidyapeeth (Deemed to be University), Karad, Maharashtra, India, from January 25, 2026 to May 15, 2026. Ethical approval was obtained from the Institutional Ethics Committee (IEC), Krishna Vishwa Vidyapeeth (Deemed to be University), Karad, Maharashtra, India (Protocol No. 371/2025-2026; Ref. No. KVV/IEC/02/2026; approval date: January 22, 2026). Written informed consent was obtained from all the participants before participation.

The sample size was calculated using the formula:

$n = \frac{2\left(Z_{\alpha}/2 + Z_{\beta}\right)^2\sigma^2}{d^2}$

where n represents the required sample size per group; Z_α_/2 was taken as 1.96, corresponding to a two-sided 95% confidence level; Z_β_ was taken as 0.84, corresponding to 80% study power; σ² represents the pooled variance of the two comparison groups (98.125), calculated from the standard deviations of 11.5 and 8.0; and d represents the expected mean difference between the two groups (10°). Substituting these values into the formula resulted in a calculated sample size of approximately 15.39 participants per group, which was rounded up to 16 participants per group. Considering an anticipated 10% dropout rate, the sample size was increased to 18 participants per group, resulting in a planned total sample size of 36 participants.

A total of 36 individuals were assessed for eligibility; six were excluded before randomization (two did not meet the inclusion criteria, two declined to participate, and two were excluded for other reasons). The remaining 30 participants were randomly allocated in a 1:1 ratio to the experimental group (n=15) and control group (n=15) using the chit method. Following allocation, six participants were lost to follow-up or excluded (four due to lack of operative reports and two due to personal reasons/transportation issues), resulting in 24 participants completing the study, with 12 participants in each group. Due to the nature of the intervention, blinding of the participants and treating physiotherapists was not feasible, and the outcome assessor was not blinded to group allocation. The chosen sample size was considered appropriate to ensure reliable statistical analysis and to effectively evaluate the potential differences in knee range of motion, pain levels, and functional outcomes between the two groups. Individuals aged between 20 and 60 years who had undergone surgical fixation for distal femur fracture, proximal tibia fracture, tibial plateau fracture, tibial tubercle fracture, or patellar fracture were included in the study. Participants who had completed a minimum post-operative immobilization period of six weeks and demonstrated restricted knee mobility, characterized by at least 30° of knee flexion and an extension lag of no more than 20°, were included in the study. Individuals with active infection or unhealed surgical wounds, unstable fixation or implant complications, neurological deficits affecting the lower limb, severe cognitive impairment, or inability to follow instructions were excluded.

**Figure 1:**
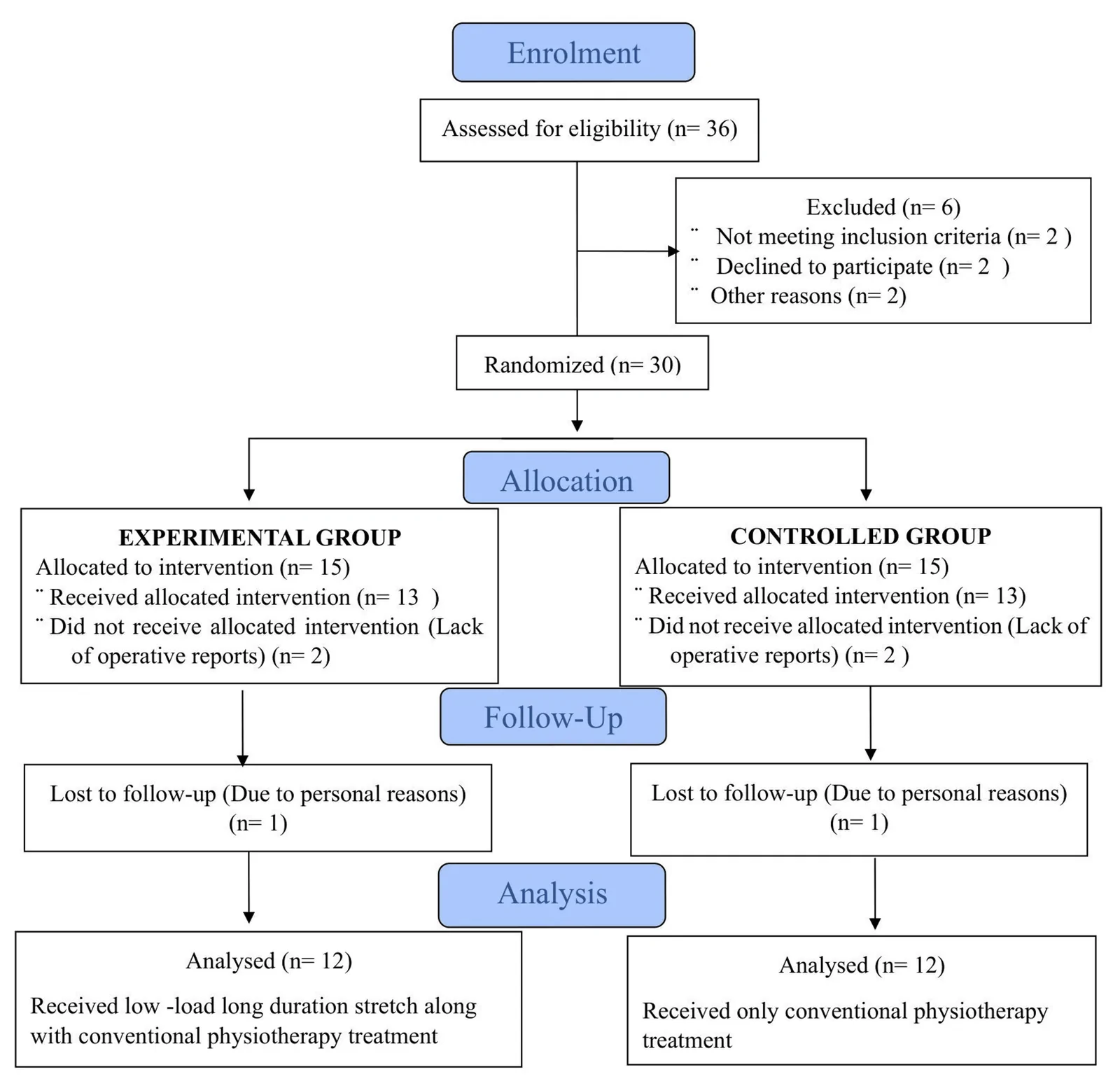
Study flowchart

Selection of outcome measures

Baseline pre-test assessment was conducted before initiation of treatment, which included measurement of pain using the Numeric Pain Rating Scale (NPRS), knee range of motion using a universal goniometer, and functional outcome using the Lysholm Knee Scoring Scale.

11-Point Numeric Pain Rating Scale (NPRS)

Pain intensity was assessed using the Numeric Pain Rating Scale (NPRS), an 11-point self-reported outcome measure ranging from 0 (no pain) to 10 (worst imaginable pain). The NPRS is a reliable and valid instrument for measuring pain intensity in individuals with musculoskeletal disorders and is sensitive to changes following therapeutic interventions [6].

Range of Motion

*Knee range of motion*: Knee flexion and extension were assessed using a universal goniometer in accordance with standardized measurement procedures [7]. Participants were positioned in the supine position on a treatment table with the lower limb adequately supported. The fulcrum of the goniometer was placed over the lateral epicondyle of the femur, the stationary arm was aligned along the lateral aspect of the femur, pointing towards the greater trochanter, and the moving arm was aligned with the lateral aspect of the fibula directed towards the lateral malleolus [8]. Measurements of knee flexion and extension were recorded in degrees. To improve the consistency of the measurements, three consecutive readings were obtained for each movement, and the mean value was used for statistical analysis.

Lysholm Knee Scoring Scale

Functional outcome was evaluated using the Lysholm Knee Scoring Scale (LKSS), a validated patient-reported questionnaire that assesses knee function across eight domains, including limp, support, locking, instability, pain, swelling, stair climbing, and squatting. The total score ranges from 0 to 100, with higher scores indicating better knee function and overall clinical outcome [9].

Intervention

Following the initial assessment, the experimental group received a structured intervention consisting of low-load, long-duration stretching to improve knee flexion and extension, along with conventional physiotherapy exercises. In contrast, the control group received only conventional physiotherapy for 30 minutes per session, three sessions per week for four weeks, consisting of active-assisted and active knee range-of-motion exercises, passive mobilization, quadriceps and hamstring isometric exercises, straight leg raises, patellar mobilization, and progressive functional exercises as tolerated, as shown in Table 1. The progression of the low-load long-duration stretch (LLLDS) protocol administered to the experimental group over the four-week intervention period is presented in Table 2.

Conventional treatment protocol for the control group

The conventional physiotherapy protocol administered to the control group is detailed in Table 1.

**Table 1:** Conventional treatment protocol for the control group ROM: Range of motion.

	Exercise programme	Duration
Warm-up	Ankle pumps and active ankle movements	10 repetitions
	Heel slides / active-assisted knee flexion	10 repetitions
	Gentle passive knee flexion and extension	10 repetitions
Week 1	Quadricep isometric sets	5 repetitions × 10-sec hold
	Hamstring isometric sets	5 repetitions × 10-sec hold
	Active-assisted knee flexion–extension	10 repetitions
	Patellar mobilization – superior/inferior and medial/lateral	1-2 minutes
	Straight leg raise, as tolerated	5 repetitions
Week 2	Quadricep isometric sets	10 repetitions×10-sec hold
	Hamstring isometric sets	10 repetitions×10-sec hold
	Active-assisted knee flexion–extension	10 repetitions
	Patellar mobilization – superior/inferior and medial/lateral	2 minutes
	Straight leg raise, as tolerated	10 repetitions
Week 3	Active knee flexion–extension ROM	10-15 repetitions
	Quadriceps strengthening	10 repetitions×10-sec hold
	Straight leg raise	10 repetitions
	Gentle knee joint mobilization	2-3 minutes
	Sit-to-stand / functional training	5-10 repetitions
Week 4	Active knee ROM with progressive end-range movement	10-15 repetitions
	Progressive quadriceps strengthening	10 repetitions × 10-sec hold
	Straight leg raise	10 repetitions
	Knee joint mobilization	2-3 minutes
	Sit-to-stand / functional weight-bearing exercises	10 repetitions

Treatment protocol for the experimental group

The four-week progression of the low-load long-duration stretch protocol administered to the experimental group, including the flexion and extension loads and treatment frequency, is presented in Table 2.

**Table 2:** Treatment protocol for the experimental group

Week	Flexion load	Extension load	Duration/Frequency
Week 1	2 kg sustained load	2 kg cuff weight	1 hour/session, once daily × 3 days/week
Week 2	2-3 kg sustained load	2-3 kg cuff weight	1 hour/session, once daily × 3 days/week
Week 3	3-4 kg sustained load	3-4 kg cuff weight	1 hour/session, once daily × 3 days/week
Week 4	4-5 kg (as tolerated, pain-free)	4-5 kg (as tolerated)	1 hour/session, once daily × 3 days/week

Following the completion of the four-week intervention period, post-intervention assessments of pain, knee range of motion, and functional outcome were performed using the same standardized assessment procedures. The collected data were tabulated and statistically analyzed to compare the pre- and post-intervention values within and between groups, thereby determining the effectiveness of low-load long-duration stretch in improving knee range of motion, reducing pain, and enhancing functional outcomes in post-operative knee conditions.

Statistical analysis

The collected data were analysed using IBM SPSS Statistics version 26.0 (IBM Corp, Armonk, NY). Descriptive statistics were expressed as mean±standard deviation (SD). The normality of the data was assessed using the Shapiro-Wilk test, and the data were found to be normally distributed. Therefore, parametric tests were used. A paired t-test was used to compare pre- and post-intervention values within each group, while an independent samples t-test was used to compare outcome measures between the experimental and control groups. A p-value of <0.05 was considered statistically significant.

## Results

The sociodemographic distribution of participants is presented in Table 3.

**Table 3:** Sociodemographic distribution of participants

Age	Total no. of participants n (%)	Experimental group n (%)	Controlled group n (%)
20-30	4 (16.7%)	2 (8.3%)	2 (8.3%)
31-40	6 (25%)	3 (12.5%)	3 (12.5%)
41-50	8 (33.3%)	4 (16.7%)	4 (16.7%)
51-60	6 (25%)	3 (12.5%)	3 (12.5%)
Total	24 (100%)	12 (50%)	12 (50%)

Table 3 presents the age-wise distribution of the study participants. The highest proportion of participants belonged to the 41-50 years age group, comprising eight participants (33.3%), followed by the 31-40 years and 51-60 years age groups, each with six participants (25.0%). The 20-30 years age group included four participants (16.7%). The distribution of participants was comparable between the experimental and control groups, with each group containing an equal number of participants across all age categories, indicating baseline homogeneity with respect to age.

The distribution of participants according to the type of fracture is presented in Table 4.

**Table 4:** Distribution of participants according to the type of fracture

Type of Fracture	Total Participants, n (%)	Experimental Group, n (%)	Control Group, n (%)
Distal femur fracture	5 (20.8%)	2 (8.3%)	3 (12.5%)
Proximal tibia fracture	5 (20.8%)	3 (12.5%)	2 (8.3%)
Tibial plateau fracture	6 (25%)	3 (12.5%)	3 (12.5%)
Patellar fracture	4 (16.7%)	2 (8.3%)	2 (8.3%)
Tibial tubercle fracture	4 (16.7%)	2 (8.3%)	2 (8.3%)
Total	24 (100%)	12 (50%)	12 (50%)

Table 4 illustrates the distribution of participants according to the type of fracture. Tibial plateau fracture was the most common injury, affecting six participants (25.0%), followed by distal femur fracture and proximal tibia fracture, each involving five participants (20.8%). Patellar fracture and tibial tubercle fracture were each observed in four participants (16.7%). The distribution of fracture types was comparable between the experimental and control groups, indicating baseline homogeneity and minimizing selection bias.

The distribution of participants according to immobilization period is presented in Table 5.

**Table 5:** Distribution of participants according to immobilization period

Immobilization Period	Total Participants, n (%)	Experimental Group, n (%)	Control Group, n (%)
6 weeks	10 (41.7%)	5 (20.8%)	5 (20.8%)
8 weeks	8 (33.3%)	4 (16.7%)	4 (16.7%)
>8 weeks	6 (25%)	3 (12.5%)	3 (12.5%)
Total	24 (100%)	12 (50%)	12 (50%)

Table 5 presents the distribution of participants according to the duration of postoperative immobilization. Ten participants (41.7%) had an immobilization period of six weeks, eight participants (33.3%) had an immobilization period of eight weeks, and six participants (25.0%) had an immobilization period of more than eight weeks. An equal distribution of participants was observed between the experimental and control groups across all immobilization periods, indicating comparable baseline characteristics.

The week-wise comparison of knee flexion range of motion between groups is presented in Table 6.

**Table 6:** Week-wise comparison of knee flexion range of motion between groups Values are expressed as Mean±SD. A between-group comparison was performed using an independent samples t-test.

Week	Experimental Mean±SD	Controlled Mean±SD	T value	P value
Week 1	38.91±3.17	35.75±2.95	2.547	0.0187
Week 2	63.66±3.72	60.25±3.81	2.219	0.0371
Week 3	87.25±4.02	83.08±3.96	2.555	0.0181
Week 4	106.33±2.93	103.50±2.68	2.470	0.0217

Table 6 demonstrates the week-wise comparison of knee flexion range of motion between the experimental and control groups over the four-week intervention period. Both groups showed progressive improvement in knee flexion throughout the study. However, the experimental group consistently demonstrated higher mean knee flexion values than the control group at each assessment. The between-group differences were statistically significant in Week 1 (p=0.0187), Week 2 (p=0.0371), Week 3 (p=0.0181), and Week 4 (p=0.0217), suggesting that the addition of low-load long-duration stretching resulted in greater improvement in knee flexion than conventional physiotherapy alone.

The comparison of pre- and post-intervention outcome measures within experimental and control groups using a paired t-test is presented in Table 7.

**Table 7:** Comparison of pre- and post-intervention outcome measures within experimental and control groups using a paired t-test

Outcome	Group	Pre-Mean±SD	Post Mean±SD	T value	P value
NPRS	Experimental	7.33±1.07	2.10±0.82	11.24	<0.0001
	Control	7.83±1.11	4.20±0.90	9.87	<0.0001
Range of Motion	Experimental	30.58 ± 3.52	106.33±2.93	41.20	<0.0001
	Control	31.58±3.42	103.50±2.68	34.75	<0.0001
Lysholm Score	Experimental	45.66±2.90	88.75±6.40	10.12	<0.0001
	Control	43.83±3.01	84.10±6.85	8.96	<0.0001

Table 7 shows the within-group comparison of pre- and post-intervention outcome measures. Both the experimental and control groups demonstrated statistically significant improvements in pain intensity, knee range of motion, and functional outcomes following the intervention (p<0.0001). The experimental group exhibited a greater reduction in pain and a larger improvement in knee range of motion and Lysholm Knee Scoring Scale scores compared with the control group, indicating the additional benefit of low-load long-duration stretching.

The comparison of post-treatment NPRS, knee range of motion, and Lysholm score between experimental and control groups are presented in Table 8.

**Table 8:** Comparison of post-treatment NPRS, knee range of motion, and Lysholm score between experimental and control groups Values are expressed as mean±SD. Between-group comparison was performed using the independent sample t-test. NPRS Numerical Pain Rating Scale.

Outcome	Experimental Post Mean±SD	Controlled Post Mean±SD	T value	P value
NPRS	2.10±0.82	4.20±0.90	2.62	0.015
Range of Motion	106.33±2.93	103.50±2.68	2.47	0.021
Lysholm Score	88.75±6.40	84.10±6.85	2.18	0.039

Table 8 compares the post-intervention outcomes between the experimental and control groups. The experimental group demonstrated significantly lower pain scores (NPRS, p=0.015), greater knee range of motion (p=0.021), and higher Lysholm Knee Scoring Scale scores (p=0.039) than the control group. These findings indicate that low-load long-duration stretching combined with conventional physiotherapy was more effective than conventional physiotherapy alone in improving pain, knee mobility, and functional outcomes.

## Discussion

The primary objective of the present study was to evaluate the effectiveness of LLLDS in improving pain, knee range of motion, and functional outcomes among individuals experiencing post-operative knee stiffness following surgical fixation of fractures around the knee joint. Post-operative knee stiffness is a common complication that can adversely affect joint mobility, delay rehabilitation, and limit the performance of daily functional activities. Therefore, identifying effective rehabilitation strategies is essential for promoting optimal recovery. The study included participants with restricted knee mobility and associated functional impairments after completing the required period of post-operative immobilization. The findings demonstrated significant improvements in pain intensity, knee range of motion, and functional performance following the intervention. Participants who received LLLDS in combination with conventional physiotherapy exhibited greater improvements than those who received conventional physiotherapy alone. These findings suggest that the incorporation of LLLDS into post-operative rehabilitation may help reduce joint stiffness, enhance knee mobility, alleviate pain, and improve overall functional recovery, thereby facilitating a more effective rehabilitation process following knee surgery.

The significant reduction in pain observed in the present study may be attributed to the biomechanical effects of prolonged low-load stretching on the muscle-tendon unit. Sustained stretching promotes the viscoelastic properties of soft tissues through mechanisms such as stress relaxation and creep, resulting in a gradual decrease in passive tissue tension and stiffness. As the resistance offered by the surrounding soft tissues decreases, excessive stress on pain-sensitive periarticular structures is reduced, allowing joint movements to be performed more comfortably. These physiological adaptations may explain the marked improvement in pain levels observed among participants who received low-load long-duration stretching in the present study, supporting its effectiveness as an adjunct to conventional physiotherapy [10].

Similar findings have been reported by Magnusson et al., who observed a significant reduction in passive torque during prolonged stretching without any corresponding increase in muscle activation. Their findings suggest that the decrease in pain and discomfort is primarily related to reduced mechanical resistance of the soft tissues rather than changes in neuromuscular activity [11]. The findings of the present study are consistent with this mechanism. Participants in the experimental group who received LLLDS demonstrated a greater reduction in pain than those in the control group, indicating that sustained end-range stretching may have promoted favourable viscoelastic changes in the periarticular soft tissues. Although participants in the control group also experienced pain relief following conventional physiotherapy, the absence of prolonged sustained stretching may have limited the extent of pain reduction observed in comparison with the experimental group.

The significant improvement in knee range of motion observed in the present study may be attributed to the concept of Total End Range Time (TERT), which emphasizes the importance of maintaining a joint at its maximum available end range for a prolonged period. According to Flowers and LaStayo, greater improvements in passive joint mobility are achieved when the duration of sustained end-range positioning is increased, demonstrating a significant positive effect on range of motion (p<0.005) [12]. Sustained low-load stretching allows gradual elongation and remodelling of periarticular connective tissues through viscoelastic adaptation, thereby decreasing soft tissue stiffness and increasing joint extensibility. The superior improvement in knee flexion range of motion observed in the experimental group of the present study is consistent with this mechanism, suggesting that prolonged end-range stretching, when combined with conventional physiotherapy, is more effective in restoring joint mobility than conventional physiotherapy alone.

The improvement in functional outcomes observed in the present study, as reflected by the higher Lysholm Knee Scoring Scale scores, may be attributed to the increase in knee joint mobility achieved through LLLDS. Improved range of motion enables individuals to perform functional activities such as walking, stair climbing, squatting, and other daily tasks with greater ease and less discomfort. Similar findings have been reported by Furia et al. [13], who demonstrated that prolonged passive stretching administered through dynamic splinting was significantly associated with improved joint range of motion in the lower extremity. The authors suggested that maintaining a joint at its end range for extended periods facilitates gradual elongation of periarticular connective tissues, reduces joint contracture, and enhances overall functional performance. These findings are consistent with the results of the present study, in which participants who received LLLDS in combination with conventional physiotherapy demonstrated greater functional improvement than those treated with conventional physiotherapy alone, indicating the beneficial role of sustained stretching in post-operative knee rehabilitation. These findings align with those of Furia et al. [13], demonstrating that prolonged end-range loading through sustained stretching can improve lower-extremity range of motion, reduce contracture, and enhance functional outcomes compared with conventional physiotherapy alone. Enhanced joint mobility and pain relief likely enabled participants to perform activities of daily living more efficiently and with greater confidence. Although participants in the control group also exhibited improvements in functional outcomes following conventional physiotherapy, the magnitude of improvement was comparatively lower than that observed in the experimental group. This difference may be attributed to the absence of sustained low-load stretching, which limits the prolonged end-range loading necessary for optimal soft tissue adaptation. Overall, the findings of the present study suggest that incorporating LLLDS into conventional rehabilitation programmes may provide additional benefits in restoring functional capacity and facilitating recovery in individuals with post-operative knee conditions [13].

Clinicians should maintain a broad differential diagnosis when evaluating patients with persistent post-operative knee pain or functional deficits. Although postoperative stiffness may commonly result from musculoskeletal restrictions, persistent or atypical symptoms should prompt consideration of alternative neurological or spinal causes. A comprehensive history and musculoskeletal and neurological examination may help identify atypical presentations and facilitate timely referral for further investigation when indicated [14,15].

In summary, the results of the present study indicate that incorporating low-load long-duration stretching alongside conventional physiotherapy contributes meaningfully to recovery in post-operative knee conditions. Participants in the experimental group experienced greater pain relief, improved knee flexion range, and enhanced functional performance compared to those receiving conventional therapy alone. The sustained and gentle nature of the stretch likely facilitated gradual soft tissue adaptation, reduced joint stiffness, and improved movement confidence. Together, these outcomes suggest that low-load long-duration stretching can serve as an effective and practical adjunct in post-operative rehabilitation programs, supporting better mobility, functional independence, and overall recovery.

Limitations

The study was conducted at a single centre with a relatively small final sample size and participant dropouts, which may limit the generalizability of the findings and introduce potential attrition bias. The short study duration also prevented long-term follow-up. Future studies with larger, multicenter samples, improved participant retention, and longer follow-up periods are recommended to confirm the findings and evaluate the long-term effects of LLLDS.

Strengths

The study addresses the common clinical problem of post-operative knee stiffness and evaluates a simple, safe, and cost-effective intervention. Standardized and reliable outcome measures were used to assess pain, knee range of motion, and functional improvement.

Future recommendations

Future research should include larger sample sizes and longer follow-up periods to assess long-term effects. Comparative studies with other rehabilitation techniques and inclusion of additional functional outcome measures are recommended for more comprehensive findings.

## Conclusions

Post-operative knee stiffness can significantly delay recovery and limit functional independence following surgical fixation of knee osseous injuries. The findings of this study demonstrate that low-load long-duration stretching is an effective and safe intervention for reducing pain, improving knee range of motion, and enhancing functional performance. Incorporating sustained, gentle stretching into post-operative rehabilitation can promote better tissue flexibility and joint mobility, ultimately supporting a smoother and more efficient recovery. Incorporating this technique into routine physiotherapy rehabilitation can help clinicians optimize treatment outcomes, prevent long-term stiffness, and facilitate a faster return to daily and functional activities for individuals recovering from knee surgery.
